# Graphlet signature-based scoring method to estimate protein–ligand binding affinity

**DOI:** 10.1098/rsos.140306

**Published:** 2014-12-10

**Authors:** Omkar Singh, Kunal Sawariya, Polamarasetty Aparoy

**Affiliations:** Centre for Computational Biology and Bioinformatics, Central University of Himachal Pradesh, Dharamshala, Himachal Pradesh 176215, India

**Keywords:** graphlet signature, interaction network, docking, binding affinity

## Abstract

Over the years, various computational methodologies have been developed to understand and quantify receptor–ligand interactions. Protein–ligand interactions can also be explained in the form of a network and its properties. The ligand binding at the protein-active site is stabilized by formation of new interactions like hydrogen bond, hydrophobic and ionic. These non-covalent interactions when considered as links cause non-isomorphic sub-graphs in the residue interaction network. This study aims to investigate the relationship between these induced sub-graphs and ligand activity. Graphlet signature-based analysis of networks has been applied in various biological problems; the focus of this work is to analyse protein–ligand interactions in terms of neighbourhood connectivity and to develop a method in which the information from residue interaction networks, i.e. graphlet signatures, can be applied to quantify ligand affinity. A scoring method was developed, which depicts the variability in signatures adopted by different amino acids during inhibitor binding, and was termed as GSUS (graphlet signature uniqueness score). The score is specific for every individual inhibitor. Two well-known drug targets, COX-2 and CA-II and their inhibitors, were considered to assess the method. Residue interaction networks of COX-2 and CA-II with their respective inhibitors were used. Only hydrogen bond network was considered to calculate GSUS and quantify protein–ligand interaction in terms of graphlet signatures. The correlation of the GSUS with pIC_50_ was consistent in both proteins and better in comparison to the Autodock results. The GSUS scoring method was better in activity prediction of molecules with similar structure and diverse activity and vice versa. This study can be a major platform in developing approaches that can be used alone or together with existing methods to predict ligand affinity from protein–ligand complexes.

## 2. Introduction

Understanding and quantifying receptor–ligand interactions forms the core of computer-aided drug discovery methods [1]. One such method, molecular docking, has been widely used owing to its high speed and performance. The approximations in the scoring methods are one of the limitations of these docking programs. Recently, to improve the performance of virtual screening experiments, approaches like free energy perturbation methods, pharmacophore modelling, post docking consensus scoring/tuned scoring, etc. are used in combination with docking methods [2]. There is a need for development of more such methods that can improve the authenticity of virtual screening findings when used alone or together with the existing methods.

System biology is an emerging discipline used to analyse and interpret various kinds of biological networks [3]. Various graph theory methods, especially residue interaction networks, have been extensively used to analyse protein structure and dynamics [4]. In the residue interaction network, amino acids (AAs) are represented as nodes and edges represent the interactions among them. Reports suggest that the protein–ligand interactions can also be explained in the form of network. The impact of a ligand binding on protein network can be measured in terms of its network properties and it has substantial effects on the closeness centrality of network [5].

The purpose of this study was to analyse the changes in local connectivity of each node (active site residues) present in protein-active site after ligand binding in the residue interaction networks and to relate these changes to compound activity. Each node present in protein-active site has its own neighbourhood and local connectivity. Binding of ligand/substrate with active site residues is mediated by the creation of new interactions. These new interactions will change the local connectivity and neighbourhood of the active site residues and induce non-isomorphic sub-graphs in the protein-active site with respect to an active site residue. Small connected non-isomorphic induced sub-graphs of large network are termed as graphlets [6]. Graphlet signature-based analysis of biological networks has been successfully applied extensively in various biological problems [7,8]. The importance of graphlet signatures in networks led to the development of combinatorial approaches for graphlet counting [9]. This study focuses on the identification of induced sub-graphs in the protein-active site after ligand binding employing graphlet signature-based analysis of residue interaction networks and their application to estimate binding affinity of various ligands.

## 3. Material and methods

The aim of the present study was to evaluate the efficiency of graphlet signatures in inhibitor activity prediction. For this study two well-known drug targets, COX-2 and CA-II, were considered. Thirty inhibitors for each of the targets were chosen randomly. The crystal structures of CA-II complexed with various inhibitors have been obtained from the Protein Data Bank (PDB) [10]. Similarly, COX-2 crystal structure co-complexed with Celecoxib was selected for the studies [11].

For the inhibitors which are not co-crystallized with these enzymes, docking procedure was employed to identify the favourable conformations at the active site. Structures in PDB, 2POU and 3LN1 were employed for docking studies with CA-II and COX-2, respectively. Prior to docking, all the potential ligands were prepared in DG-AMMOS [12] using AMMP force field sp4. Autodock program was used for docking [13]. During the docking process, maximum number of conformer generation was set to 100 and other parameters were set to default values.

**Figure 1.**
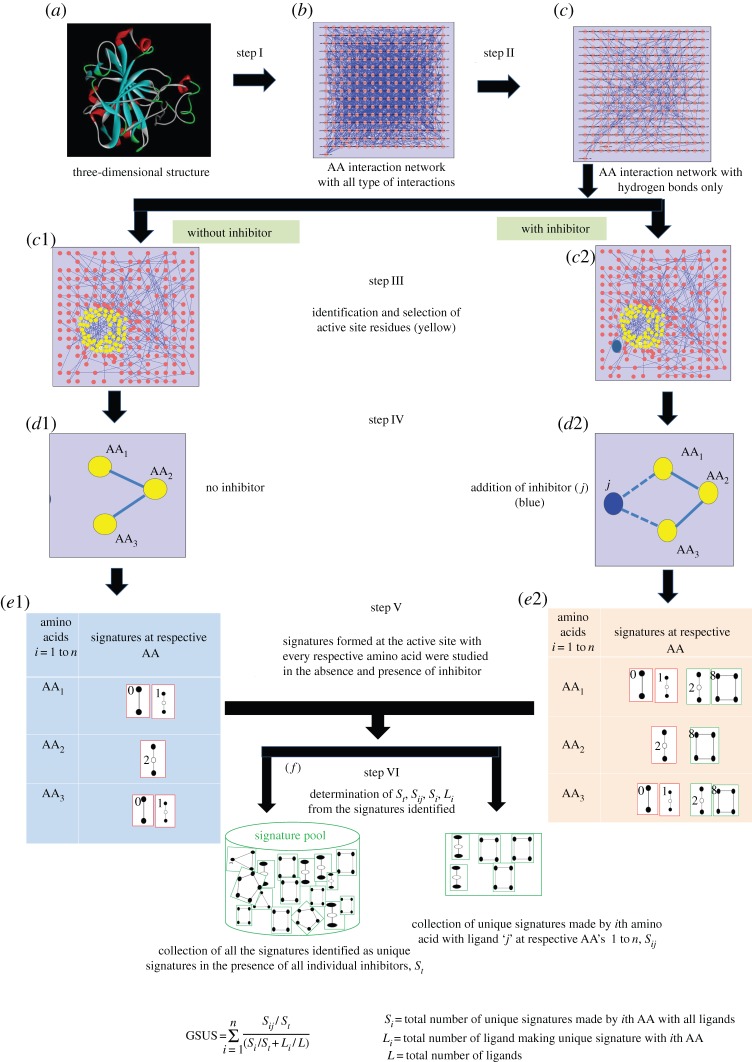
Flowchart depicting the work plan: (*a*) crystal structure of CA-II retrieved from PDB; (*b*) residue interaction network of the PDB structure with all types of non-covalent interactions generated by RING server; (*c*) residual interaction network of the hydrogen bond interactions from RINanalyzer. Identification and selection of active site residue (yellow) and analysis of graphlet signature by using GRAPHLET COUNTER in the absence of ligand (*c*1–*e*1) and in the presence of ligand (*c*2–*e*2) at the active site (yellow) and (*f*) extraction of new signatures and computation of GSUS.

In this study, residue interaction networks for COX-2 (3LN1) and CA-II (2POU) [11,14] were obtained from RING server [15]. Furthermore, the networks were visualized and analysed in Cytoscape [16]. The hydrogen bond pattern in protein structure was identified using the HB explore tool in RING server [17] and was analysed in RINalyzer [18]. Further, graphlet counter was used to examine the signature patterns made by different inhibitors with the active-site residues [19] (figure 1). Ligand binding at the protein-active site is stabilized by the formation of non-covalent interactions. Hydrogen bond interactions play a major role in proteins [20]. The residues present in protein-active site have hydrogen bond connectivity with their neighbours and create a local hydrogen bond network. Ligand binding leads to change in the graphlet signatures of the residues at the active site. In this study, only the hydrogen bond network of the protein was considered and the effects of ligand binding on the signatures at the active site were studied. The signature analysis method that provides an opportunity to analyse the residues that are not in direct contact with inhibitors and are in secondary shell of active site is also considered in this approach.

The graphlet signatures corresponding to every AA in the active site were analysed before and after ligand binding. Unique signatures, i.e. the signatures that are formed at the active site only after ligand binding were identified. These are the signatures which exist only after ligand binding and are non-existent in apoprotein structure.

A pool of all the unique signatures formed by the 30 inhibitors with respect to the residues present in protein-active site was created. Furthermore, the information from these graphlet signatures was employed to quantify affinity of every inhibitor in the dataset. Uniqueness score for each inhibitor was measured in terms of variability in signatures adopted by different AAs during inhibitor binding. The uniqueness at these signatures was quantified as follows:

3.1 $$\text{GSUS}=\sum_{i=1}^{n}\frac{S_{ij}/S_{t}}{\left(S_{i}/S_{t}+L_{i}/L\right)},$$

where

GSUS = graphlet signature uniqueness score for ligand *j*;
$S_{ij}=\sum_{k=1}^{73}\max(0,X_{ijk}-X_{i^{*}k})$, where *X*_*ijk*_−*X*_i*k_={1 if *X*_*ijk*_≠*X*_i*k_,else 0}, *X*_i*k_ represent the total number of signature in absence of ligand with respect to *i*th AA, *X*_*ijk*_ represent the total number of signature in presence of ligand with respect to *i*th AA in particular orbit *k*, and *S*_*ij*_ represents the number of unique signatures made by *i*th AA with inhibitor *j*;
*S*_*t*_=total number of unique signatures made by all the ligands with all AAs (signature pool);
$S_{i}=\sum_{j=1}^{L}S_{ij}$ and it represents the total number of unique signatures made by *i*th AA with all the ligands;
$L_{i}=\sum_{j=1}^{L}\min(S_{ij},1)$ and it represents the number of ligands forming unique signatures with *i*th AA; and
*L* = total number of ligands used in the dataset.

The correlation between the biological activity and predicted activity (GSUS and Autodock score with default settings) for the compounds in the dataset was performed using Pearson’s correlation coefficient. The pair-wise diversity of the compound dataset was measured. The dependency of the method on diversity was illustrated to check if the methods hold good for various scaffolds. The similarity between each pair of molecules was measured using Tanimoto coefficient in OpenBabel [21].

## 4. Results and discussion

Studies on the application of network properties to differentiate ligand selectivity have been reported [22]. Graphlet signature methods have been successfully applied to identify functional residues in protein-active site and ligand-binding site predictions [23,24]. This study focuses on the knowledge of induced sub-graphs in protein-active site and their application to estimate and quantify protein–ligand interactions is novel and promising.

### 4.1 Studies on COX-2

RING server was used to generate the residue interaction network of COX-2. All the AAs within 10 Å radius from the centre of the active site were selected (electronic supplementary material, table S1). From the hydrogen bond interaction networks formed, we analysed signatures with varying orbits present at each selected residue. Collectively, these were termed as native signatures of active site as they are present in the native structure of protein, i.e. non-inhibitor bound form. To analyse the hydrogen bond interactions between inhibitor and enzyme, the complexes of 30 inhibitors with COX-2 were used. The respective hydrogen bonds formed by each of the inhibitors at the active site were identified (electronic supplementary material, table S2). Prior to the graphlet signature analysis, the individual inhibitors were added to the protein–hydrogen bond network manually and the contacts were created between the inhibitor and the respective hydrogen bond forming AAs. The graphlet signatures of each individual inhibitor with the active site AAs were studied and unique signatures, i.e. the new signature formed from non-existence at each of the AAs, were identified.

**Figure 2.**
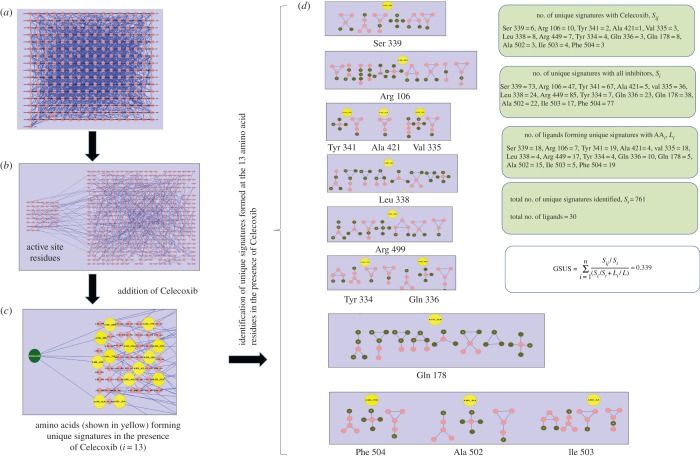
Computation of GSUS of Celecoxib with COX-2: (*a*) AA interaction network; (*b*) selection of active site residues in hydrogen bond network; (*c*) Celecoxib induces unique graphlet signatures with respect to the AAs present in the active site (yellow) and (*d*) various signature parameters formed with respect to individual AAs.

In some cases, it was observed that the same AA was involved in signatures with different orbits more than once. Each of such situations was counted as unique signatures. All the unique signatures formed with each of the selected AAs by every inhibitor were identified as listed in electronic supplementary material, table S3. The total number of unique signatures for all the inhibitors with all the AAs was found to be 761. This collection was termed as signature pool, *S*_*t*_. All the quantified features were further applied in the calculation of GSUS for each inhibitor using equation (3.1) (table 1). Calculation strategy used for Celecoxib is shown in figure 2 with all the graphlet signature details. Valdecoxib had the highest GSUS of 0.756, and lowest score was 0.0064 for Etodalac. Most of the active molecules were scored high and least active were scored low. For all the 30 molecules, correlation coefficient was 0.55. The correlation results clearly indicate positive association between biological activity and GSUS. The correlation of the GSUS with pIC_50_ was almost the same as the correlation of Autodock scores with pIC_50_, which was −0.55. The major challenges for *in silico* binding affinity prediction methods are to differentiate structurally similar molecules with different activities and structurally diverse molecules with similar activity. To check the efficiency of GSUS method in such cases, subset of compounds were made based on structure similarity (greater than 0.7) quantified by Tanimoto coefficient (electronic supplementary material, table S5). Dichlofenac and Lumiracoxib have high similarity in structure but there is 700-fold difference in their pIC_50_ values against COX-2. Similarly, four pairs of compounds, Ibuprofen/Naproxen, Piroxicam/Meloxicam, SC-560/SC58125 and flufenamic acid/mefenamic acid have high structural similarity and diverse pIC_50_ values. GSUS method was more accurate in differentiating active and inactive molecules in the subsets. Autodock was unable to distinguish the activities of the molecules with similar structures.

**Table 1.** Estimation of binding affinity of COX-2 inhibitors.

no.	drug	IC_50_ (nM)	pIC_50_	GSUS	Autodock score
1	6-methylnaphthylacetic acid	80 000	4.09691	0.16908121	−7.09
2	Piroxicam	70 000	4.154902	0.00913255	−8.13
3	Etodalac	60 000	4.221849	0.00639931	−7.49
4	Ibuprofen	40 000	4.39794	0.01738897	−7.04
5	flufenamic acid	20 000	4.69897	0.10528901	−7.1
6	ETYA	15 000	4.823909	0.02308672	−7.17
7	BW755C	10 000	5	0.07490419	−5.71
8	Lumiracoxib	7000	5.154902	0.02972949	−7.68
9	SC-560	6300	5.200659	0.0237849	−8.74
10	Etoricoxib	5000	5.30103	0.01738897	−11.16
11	Fenclofenac	4000	5.39794	0.09343407	−8.26
12	Ketoprofen	2500	5.60206	0.02308673	−8.71
13	Suprofen	2000	5.69897	0.01738897	−8.4
14	Naproxen	2000	5.69897	0.05585896	−7.15
15	Flurbiprofen	500	6.30103	0.01335906	−7.58
16	Nimuslide	500	6.30103	0.06857699	−8.98
17	Rofecoxib	500	6.30103	0.22399585	−10.79
18	Meloxicam	400	6.39794	0.49026752	−8.27
19	Licofelon	370	6.431798	0.03997682	−9.57
20	SC-58125	300	6.522879	0.01738897	−9.99
21	mefenamic acid	300	6.522879	0.018128	−7.56
22	Flosulide	130	6.886057	0.23717307	−8.85
23	CHEMBL257539	100	7	0.1167376	−8.65
24	Indisulam	100	7	0.12526685	−9.46
25	niflumic acid	100	7	0.23149516	−6.67
26	NS398	81	7.091515	0.05656345	−9.1
27	Celecoxib	50	7.30103	0.40196448	−10.35
28	Dichlofenac	9.4	8.02	0.44306776	−8.32
29	DUP-697	8.7	8.060481	0.01738894	−11.22
30	Valdecoxib	5	8.30103	0.7564579	−10.54

The performance of scoring method was also assessed for distinguishing pairs of inhibitors with very low structural similarity and high activity similarity (electronic supplementary material, table S6). COX-2 inhibitor pairs indomethacin and niflumic acid, SC58125 and mefenamic acid, Flurbinprofen/Nimesulide, CHEMBL257539/indomethacin, etc. show very low structural similarity but their activity against COX-2 is almost the same. GSUS method was more accurate in the activity prediction of these molecules and the results show clearly that GSUS is more efficient in differentiating similar structure molecules with varied activity and diverse structure molecules with similar activity.

### 4.2 Studies on CA-II

The unique signature selection was performed using the same procedure as we used in COX-2. The total number of unique signatures was found to be 1201 collectively for all the inhibitors (electronic supplementary material, table S4). All the quantified features were further applied in the calculation of GSUS for each inhibitor using equation (1) and it was observed that topiramate had the highest GSUS of 0.3, and lowest value was 0.002 for 2-hydroxy-3-methylbenzoic acid (table 2). Correlation coefficient has been calculated for pIC_50_ value and GSUS. Correlation coefficient was 0.40 for all the 30 molecules and was significant at the 0.05 level (two tailed). In the dataset of CA-II inhibitors considered, three pairs of inhibitors, 2-aminobenzenesulfonamide/2-hydrazinylbenzenesulfonamide, 2-hydroxy-3-methylbenzoic acid/4-amino-2-hydroxybenzoic acid and 4-amino-6-chlorobenzene-1,3-disulfonamide/dichlorophenamide, showed high structural similarity of Tanimoto coefficient greater than 0.7 with activity difference of twofold, sixfold and twofold, respectively (electronic supplementary material, table S7). The performance of GSUS was better in distinguishing the activities of these molecules with similar structure.

**Table 2.** Estimation of binding affinity of CA-II inhibitors.

no.	drug	IC_50_ (nM)	pIC_50_	GSUS	Autodock score
1	2-hydroxy-3-methylbenzoic acid	4 700 000	2.33	0.002865	−5.08
2	4-amino-2-hydroxybenzoic acid	750 000	3.12	0.044757	−4.6
3	2-hydroxy-5-sulfobenzoic acid	290 000	3.54	0.047693	−5.87
4	saccharin	5950	5.225483	0.009956	−4.32
5	(E)-6-oxo-3-(2-(4-(*N*-(pyridin-2-yl)sulfamoyl)phenyl)hydrazono) cyclohexa-1,4-dienecarboxylic acid	4490	5.35	0.177222	−6.97
6	2-hydroxy-3,5-dinitrobenzoic acid	2800	5.55	0.016072	−5.33
7	3-(4-sulfamoylphenyl)propanoic acid	495	6.305395	0.044916	−6.18
8	2-aminobenzenesulfonamide	295	6.530178	0.02644	−5.75
9	4-sulfamoylbenzoic acid	133	6.876148	0.105051	−5.58
10	2-hydrazinylbenzenesulfonamide	124	6.906578	0.098203	−6.21
11	4-amino-6-chlorobenzene-1,3-disulfonamide	75	7.124939	0.089529	−7.07
12	4-amino-6-(trifluoromethyl)benzene-1,3-disulfonamide	63	7.200659	0.046369	−6.66
13	4-amino-3-fluorobenzenesulfonamide	60	7.221849	0.02644	−5.24
14	4-amino-*N*-(4-sulfamoylphenethyl) benzenesulfonamide	50	7.30103	0.148955	−7.65
15	methazolamide	50	7.30103	0.114469	−4.79
16	4-amino-*N*-(4-sulfamoylbenzyl)benzenesulfonamide	46	7.337242	0.137639	−7.15
17	sulpiride	40	7.39794	0.097432	−6.77
18	dichlorophenamide	38	7.420216	0.076751	−5.38
19	zonisamide	35	7.455932	0.055204	−6.95
20	4-((2-aminopyrimidin-4-yl)amino)benzenesulfonamide	33	7.481486	0.162283	−5.79
21	Celecoxib	21	7.677781	0.088067	−6.56
22	5-imino-4-methyl-4,5-dihydro-1,3,4-thiadiazole-2-sulfonamide	19	7.721246	0.095185	−5.35
23	indisulam	15	7.823909	0.082407	−6.83
24	acetazolamide	12	7.920819	0.0338	−4.66
25	topiramate	10	8	0.3007	−4.86
26	sulthiame	9	8.045757	0.03563	−4.39
27	benzolamide	9	8.045757	0.076824	−5.06
28	dorzolamide	9	8.045757	0.110511	−5.69
29	ethoxzolamide	8	8.09691	0.16463	−5.18
30	brinzolamide	3	8.522879	0.110511	−4.53

The performance of GSUS method in CA-II was consistent with that in COX-2, unlike Autodock which showed great difference in correlation coefficient in both of these enzymes. The results in this study hint that GSUS method is promising, and it can be further improved into a more applicable and reliable method. Its performance in distinguishing activities of structurally related and unrelated compounds shows that it can be a part of virtual screening experiments employing multi-layered screening methods.

## Supplementary Material

Various results have been incorporated in the supplementary material
