# Supplementary material for: Graphlet signature-based scoring method to estimate protein–ligand binding affinity
Source: R Soc Open Sci. 2014 Dec 10;1(4):140306. doi: 10.1098/rsos.140306 (PMC4448774; doi:10.1098/rsos.140306)
Supplement: Various results have been incorporated in the supplementary material [file rsos140306supp1.pdf]

Selected 52 Amino acids In active site of COX-2	A:79:_:THR, A:78:_:LEU, A:75:_:HIS, A:517:_:LEU A:516:_:SER, A:515:_:PHE, A:513:_:ALAA:512:_:GLY,A:511:_:LEU,A:509:_:VAL A:508:_:MET,A:505:_:GLY,A:504:_:PHE,A:503:_:ILE,A:502:_:ALAA:500:_:PRO A:499:_:ARG,A:497:_:LYS,A:493:_:LEU,A:421:_:ALA,A:420:_:VAL,A:373:_:TRP A:372:_:HIS,A:371:_:TYR,A:370:_:LEU,A:369:_:THR,A:368:_:ASN,A:367:_:PHE A:366:_:GLU,A:345:_:LEU,A:344:_:LYS,A:343:_:PHE,A:341:_:TYR,A:340:_:GLY A:339:_:SER,A:338:_:LEU,A:337:_:HIS,A:336:_:GLN,A:335:_:VAL,A:334:_:TYR A:333:_:ASP,A:332:_:GLU,A:331:_:ILE,A:330:_:VAL,A:313:_:GLN,A:192:_:THR A:182:_:MET,A:178:_:GLN,A:107:_:SER,A:106:_:ARG,A:105:_:SER,A:102:_:VAL
Selected 61 Amino acids In active site of CA-II	pdb1ABC:A:97:_:TRP,pdb1ABC:A:96:_:HIS,pdb1ABC:A:95:_:PHE,pdb1ABC:A:94:_:HIS pdb1ABC:A:93:_:PHE,pdb1ABC:A:92:_:GLN,pdb1ABC:A:91:_:ILE,pdb1ABC:A:89:_:ARG pdb1ABC:A:69:_:GLU,pdb1ABC:A:68:_:VAL,pdb1ABC:A:67:_:ASN,pdb1ABC:A:66:_:PHE pdb1ABC:A:65:_:ALA,pdb1ABC:A:64:_:HIS,pdb1ABC:A:63:_:GLY,pdb1ABC:A:62:_:ASN pdb1ABC:A:61:_:ASN,pdb1ABC:A:60:_:LEU,pdb1ABC:A:30:_:PRO,pdb1ABC:A:29:_:SER pdb1ABC:A:28:_:GLN,pdb1ABC:A:245:_:TRP,pdb1ABC:A:244:_:ASN,pdb1ABC:A:211:_:VAL pdb1ABC:A:210:_:ILE,pdb1ABC:A:209:_:TRP,pdb1ABC:A:208:_:THR,pdb1ABC:A:207:_:VAL pdb1ABC:A:206:_:CYS,pdb1ABC:A:205:_:GLU,pdb1ABC:A:204:_:LEU,pdb1ABC:A:203:_:LEU pdb1ABC:A:202:_:PRO,pdb1ABC:A:201:_:PRO,pdb1ABC:A:200:_:THR,pdb1ABC:A:199:_:THR pdb1ABC:A:198:_:LEU,pdb1ABC:A:197:_:SER,pdb1ABC:A:196:_:GLY,pdb1ABC:A:146:_:ILE pdb1ABC:A:145:_:GLY,pdb1ABC:A:144:_:LEU,pdb1ABC:A:143:_:VAL,pdb1ABC:A:142:_:ALA pdb1ABC:A:141:_:LEU,pdb1ABC:A:140:_:GLY,pdb1ABC:A:136:_:GLN,pdb1ABC:A:135:_:VAL pdb1ABC:A:134:_:ALA,pdb1ABC:A:132:_:GLY,pdb1ABC:A:131:_:PHE,pdb1ABC:A:123:_:TRP pdb1ABC:A:122:_:HIS,pdb1ABC:A:121:_:VAL,pdb1ABC:A:120:_:LEU,pdb1ABC:A:119:_:HIS pdb1ABC:A:118:_:LEU,pdb1ABC:A:117:_:GLU,pdb1ABC:A:107:_:HIS,pdb1ABC:A:106:_:GLU pdb1ABC:A:105:_:SER

Table S1. All the selected amino acids of COX-2 and CA-II in 10Å radius

Name of the COX-2 Inhibitor	Structure	Hydrogen/Non-hydrogen bond Interaction	Name of CA-II Inhibitor	Structure	Hydrogen/Non-hydrogen bond Interaction
1.6methylnaphthylaceticacid	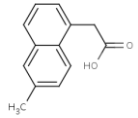	SER516,TYR371,MET508,TYR371,TRP373,VAL509,LEU338.	1. 2-aminobenzenesulfonamide		HIS119,THR199,THR200LEU198,HIS94,HIS96,TRP209,VAL121,VAL143.
2. BW755C	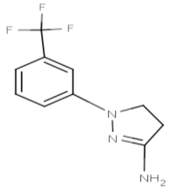	SER339,GLY512,MET508,VAL509,LEU338,TRP373,PHE504.	2. 3-(4-sulfamoyl phenyl)propan-oic acid	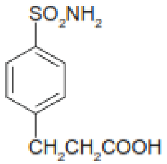	ASN67,HIS119,THR199,THR200, VAL121,LEU198, HIS64,
3. Celecoxib	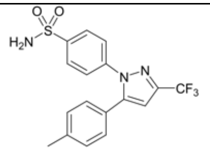	ARG499,ARG106, PHE504, GLN178,LEU338,HIS75, SER339,VAL335,VAL509,GLY512,ALA513,LEU370,MET508, LEU345,LEU517,TRP373.	3. 4-amino-3-fluorobenzenesulfonamide	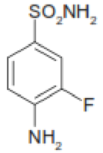	HIS119,THR199,THR200, VAL121,HIS96,HIS94,LEU198.
4. Diclofenac	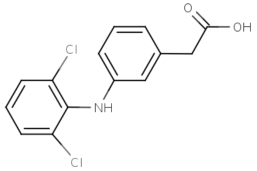	ARG106,VAL509,TYR341, MET508,GLY512, ALA513,VAL335,PHE504.	4. 4-amino-6-(trifluoromethyl) benzene-1,3-disulfonamide	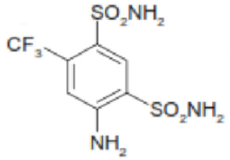	ASN67,HIS119,THR199, THR200,LEU198,GLN92, HIS64,HIS94,HIS96, TRP209,VAL121.
5. DUP-697	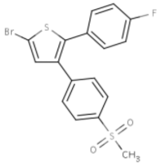	ARG499,PHE504,VAL335,SER339,VAL509, ALA513,LEU338,ALA513.	5. 4-amino-6-chlorobenzene-1,3-disulfonamide	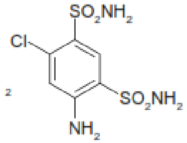	ASN67,HIS119,THR199, THR200,GLN92,HIS64, HIS94,HIS96,TRP209, VAL121,VAL143,LEU198.
6. Etodalac	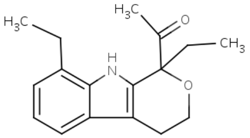	TYR341,VAL335,ALA513,LEU517,VAL102, LEU335,LEU345,VAL509,HIS75.	6. 5-imino-4-methyl-4,5-dihydro-1,3,4-thiadiazole-2-sulfonamide.	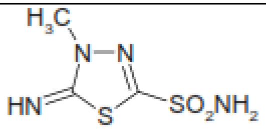	HIS119,THR199,HIS64, THR200,HIS96,

7. Etoricoxib	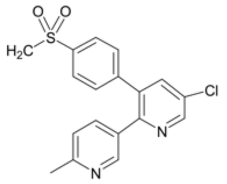	ARG499,PHE504,VAL335,SER339,VAL509, LEU370,MET508,TRP373,ALA513,LEU338.	7. 4-amino-N-(4-sulfamoylbenzyl) benzenesulfonamide	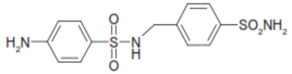	ASN62,ASN67,HIS119, THR199,PHE95,HIS64, HIS94,HIS96,TRP209, ALA65,VAL121,LEU198.
8. ETYA	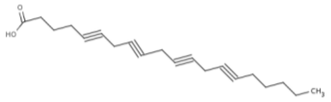	ARG499,SER339,VAL509,PHE504.	8. 4-amino-N-(4-sulfamoyl phenethyl) benzenesulfonamide	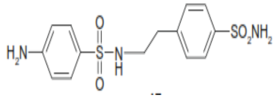	ASN62,ASN67,HIS119, THR199,THR200,PHE95, HIS64,HIS94,HIS96, TRP209,ALA65,VAL121, LEU198.
9. Flosulide	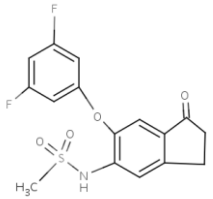	TYR341,PHE504,SER339,GLY512,TRP373, LEU338,VAL509.	9. 4-((2-amino-pyrimidin-4-yl)amino)benzenesulfonamide	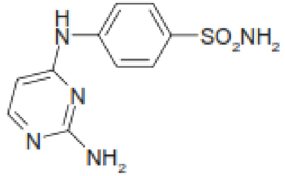	THR200,PHE95,SER197, ASN67,THR199,HIS94, HIS64,LEU198,ALA65.
10. Ibuprofen	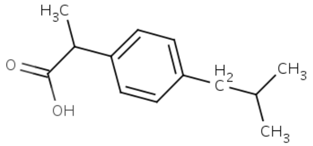	ARG499,PHE504,VAL509,ALA513,LEU338.	10. 2-hydrazinylbenze-nesulfonamide	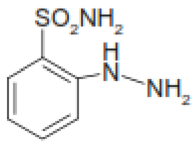	HIS119,THR199,THR200, SER197,VAL143,HIS96, HIS119,VAL121,LEU198, VAL207.
11. Indomethacin	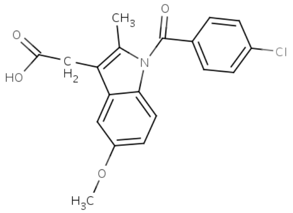	ARG106,TYR341,LEU338,VAL335,PHE504, LEU517,ALA513,LEU370,MET508,TRP373, VAL509.	11. 4-sulfamoyl-benzoic acid	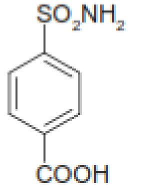	ASN67,THR199,HIS96, LEU198,THR200,HIS94, HIS96,TRP209.
12. meloxicam	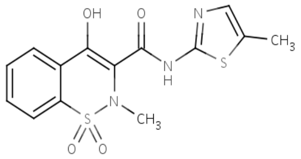	ARG499,SER339,HIS75,TYR341,MET508, VAL509,TRP373,LEU370,ALA502,LEU338.	12. Indisulam	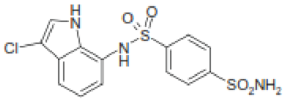	ASN67,THR199,HIS64, THR200,HIS94,LEU198.

13. Naproxen	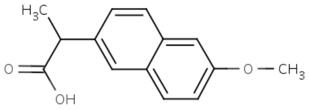	ARG106,TYR341,PHE504,VAL509.	13. Sulpiride	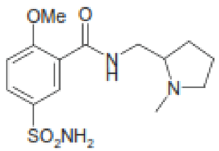	HIS119,THR199,ASN67, GLN92,HIS94,HIS96, TRP209,LEU198,VAL121.
14. Nimuslide	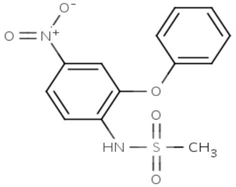	ILE503,PHE504,SER339,VAL509,TRP373, LEU338.	14. Zonisamide	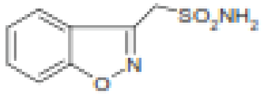	GLN92,HIS119,THR199, HIS64,HIS94,VAL143, LEU198.
15. NS 398	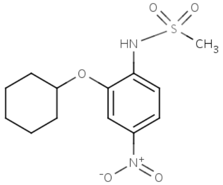	ARG499,PHE504,SER339,VAL509,LEU338, MET508,TRP373.	15. Celecoxib	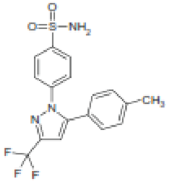	ASN67,THR199,SER197, HIS96,HIS94,TRP209, HIS64,ALA65,VAL121, LEU198.
16. Piroxicam	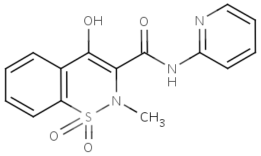	SER339,VAL509,TYR371,TRP373,MET508.	16. Topiramate	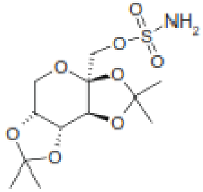	ASN62,GLN92, THR198,THR199, HIS94,LEU197 TRP208,ALA65, VAL121,HIS96,
17. Rofecoxib	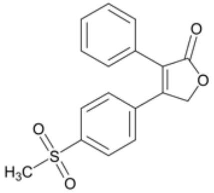	ARG499,PHE504,ALA513,SER339,VAL509, MET508,LEU338.	17. Acetazolamide	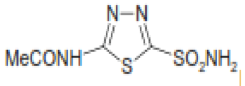	GLN92,THR199, LEU198,TRP209.

18. SC-560	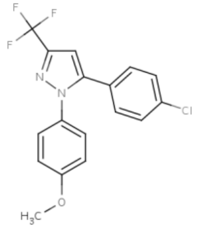	SER339,PHE504,VAL509,LEU370,MET508, VAL335,LEU345,TYR341,TRP373,VAL335, ALA513,LEU338.	18. Methazolamide	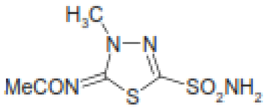	ASN67,VAL121, HIS94,HIS96, LEU198, GLN92,THR200, HIS64,THR199.
19. SC-58125	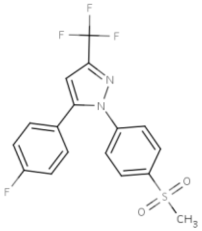	ARG499,PHE504,VAL335,SER339,VAL509, ALA513,LEU517, LEU338.	19. Ethoxzolamide	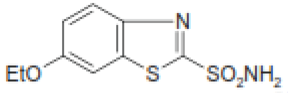	HIS119,THR199, HIS94,HIS96, LEU198,TRP209,
20. Valdecoxib	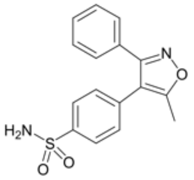	ARG499,PHE504,HIS75,SER339,GLN178, ALA513,VAL509,MET508, GLY512,VAL335, LEU338.	20. Dichlorophenamide	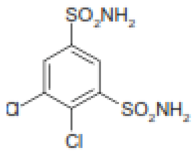	ASN67,GLN92, THR199,THR200, HIS64,TRP209, VAL121,LEU141, VAL143,LEU198, PHE131.
21. CHEMBL257539		ARG106,TYR341,SER516,ALA513,VAL335.	21. Dorzolamide	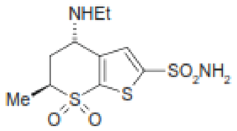	GLN92,THR199, HIS94,LEU198, PHE131,TRP209.
22. Fenclofenac	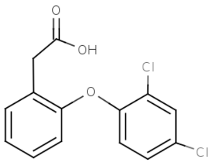	SER516,TYR371,LEU338,TYR373,VAL335, TYR341,PHE504,ALA513.	22. Brinzolamide	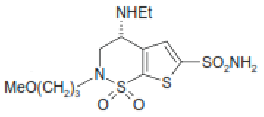	GLN92,THR199, HIS94,LEU198, TRP209,PHE131, VAL121.

23. Flufenamic acid	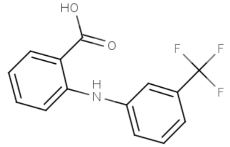	TYR341,TYR371,ARG499,GLY512,VAL509, PHE504,LEU338,ALA502.	23. Benzolamide	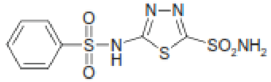	GLN92,THR199, LEU198,TRP209, HIS94,PHE131.
24. Flurbiprofen	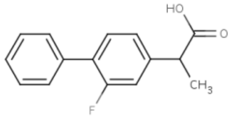	ARG106,ALA513,MET508, GLY512,VAL335, VAL509.	24. Saccharin	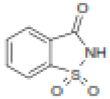	THR199,THR200, LEU198,TRP209, VAL121.
25. Ketoprofen	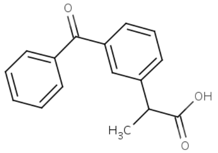	ARG499,SER339,VAL509,PHE504,LEU338.	25. Sulthiame	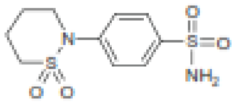	GLN92,THR199, LEU198,TRP209, HIS94,VAL121.
26. Licofelone	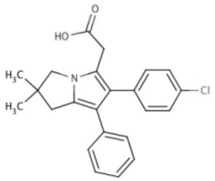	GLY512,SER516,VAL509,ALA513,LEU338, VAL102,LEU345,TYR341,TYR371,TRP373, VAL335,LEU517.	26. 2-hydroxy-3-methylbenzoic acid	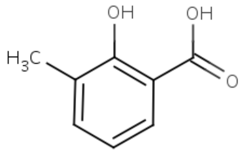	THR199, LEU198,VAL143,VAL207 TRP209, VAL121
27. Lumiracoxib		ARG106,TYR341,MET508,LEU338,VAL509, PHE504,ALA513,VAL335.	27. 4-amino-2-hydroxybenzoic acid	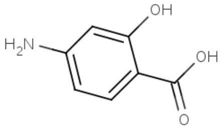	THR199, HIS96, THR200 LEU198, VAL121
28. Mefenamic acid	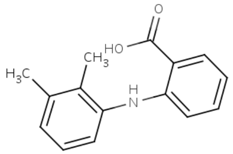	ARG499,TYR341,VAL509,VAL335,LEU338, ALA502.	28. 2-hydroxy-5-sulfobenzoic acid	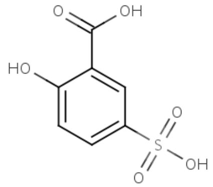	HIS119,THR199,THR200 ASN67, HIS96, HIS94

29. Niflumic acid	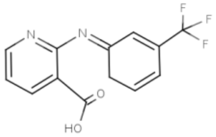	ARG499,TYR341,GLY512,GLN178,MET508, VAL509,LEU338,PHE504,VAL335,ALA502.	29. 2-hydroxy-3,5-dinitrobenzoic acid	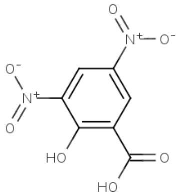	ASN67, THR199, LEU198, VAL121
30. Suprofen	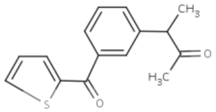	ARG499,PHE504,VAL509,TRP373,LEU338.	30. (E)-6-oxo-3-(2-(4-(N-(pyridin-2-yl)sulfamoyl)phenyl)hydrazono)cyclohexa-1,4-dienecarboxylic acid	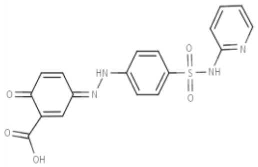	HIS119, THR199,THR200 PRO202, HIS64,HIS94,LEU198,VAL121

Table S2. COX-2 and CA-II Inhibitors with hydrogen bond (Red) and non hydrogen bond (Black) interactions.

S.No.	Inhibitor	GSUS	pIC_{50}	Active site residues and the corresponding Signature parameters identified				
				Amino Acid	S_{ij}	S_i	l_i	S_t
1.	6MMPA	0.16908121	4.09691	371Y<br>508M<br>512G<br>516S	5<br>2<br>5<br>3	17<br>34<br>59<br>43	3<br>8<br>8<br>8	761 for each Inhibitor
2.	BWCC755C	0.07490419	4.154902	335V<br>336Q<br>339S<br>341Y<br>508M<br>512G<br>516S	2<br>1<br>3<br>1<br>3<br>7<br>6	36<br>23<br>73<br>67<br>34<br>59<br>43	18<br>10<br>18<br>19<br>8<br>8<br>8	
3.	Celecoxib	0.40196448	4.221849	339S<br>106R<br>341Y<br>421A<br>335V<br>338L<br>499R<br>334Y<br>336Q<br>178Q<br>502A<br>503I<br>504F	6<br>10<br>2<br>1<br>3<br>8<br>7<br>4<br>3<br>8<br>3<br>4<br>3	73<br>47<br>67<br>5<br>36<br>24<br>85<br>7<br>23<br>38<br>22<br>17<br>77	18<br>7<br>19<br>4<br>18<br>4<br>17<br>4<br>10<br>5<br>15<br>5<br>19	
4.	Dichlofenac	0.44306776	4.39794	106R<br>335V<br>339S<br>341Y<br>505G	7<br>1<br>2<br>5<br>4	47<br>36<br>73<br>67<br>4	7<br>18<br>18<br>19<br>1	

				509V	9	9	1
5.	DUP697	0.01738894	4.69897	499R<br>502A<br>504F	4<br>1<br>4	85<br>22<br>77	17<br>15<br>19
6.	Etodalac	0.00639931	4.823909	341Y	4	67	19
7.	Etoricoxib	0.01738897	5	499R<br>502A<br>504F	4<br>1<br>4	85<br>22<br>77	17<br>15<br>19
8.	ETYA	0.02308672	5.154902	335V<br>336Q<br>339S<br>341Y<br>499R<br>502A<br>504F	2<br>1<br>3<br>1<br>2<br>1<br>1	38<br>23<br>73<br>67<br>85<br>22<br>77	18<br>10<br>18<br>19<br>17<br>15<br>19
9.	Flosulide	0.23717307	5.200659	330V<br>335V<br>336Q<br>339S<br>340Q<br>341Y<br>345L<br>499R<br>504F<br>508M<br>512G<br>516S	1<br>5<br>5<br>11<br>1<br>5<br>1<br>1<br>10<br>5<br>14<br>8	2<br>38<br>23<br>73<br>2<br>67<br>2<br>85<br>77<br>34<br>59<br>43	2<br>18<br>10<br>18<br>2<br>19<br>2<br>17<br>19<br>8<br>8<br>8

10.	Ibuprofen	0.01738897	5.30103	499R<br>502A<br>504F	4<br>1<br>4	85<br>22<br>77	17<br>15<br>19	
11.	Indisulum	0.12526685	5.39794	106R<br>178Q<br>334Y<br>335V<br>338L<br>339S<br>341Y<br>503I	7<br>4<br>1<br>1<br>5<br>2<br>5<br>1	47<br>38<br>7<br>38<br>24<br>73<br>67<br>17	7<br>5<br>4<br>18<br>4<br>18<br>19<br>5	
12.	Meloxicam	0.49026752	5.60206	330V<br>335V<br>336Q<br>339S<br>340Q<br>341Y<br>345L<br>499R<br>502A<br>504F<br>508M<br>512G<br>516S<br>75H<br>79T	1<br>5<br>5<br>12<br>1<br>6<br>1<br>10<br>3<br>3<br>14<br>6<br>3<br>12<br>5	2<br>38<br>23<br>73<br>2<br>67<br>2<br>85<br>22<br>77<br>34<br>59<br>43<br>24<br>10	2<br>18<br>10<br>18<br>2<br>19<br>2<br>17<br>15<br>19<br>8<br>8<br>8<br>2<br>2	
13.	Naproxen	0.05585896	5.69897	106R<br>335V<br>339S<br>341Y<br>499R<br>504F	7<br>1<br>2<br>5<br>1<br>5	47<br>38<br>73<br>67<br>85<br>77	7<br>18<br>18<br>19<br>17<br>19	

14.	Nimuslide	0.06857699	5.69897	178Q<br>421A<br>503I<br>504F	3<br>2<br>3<br>6	38<br>5<br>17<br>77	5<br>4<br>5<br>19	
15.	NS398	0.05656345	6.30103	335V<br>336Q<br>339S<br>341Y<br>499R<br>502A<br>504F	3<br>2<br>6<br>2<br>8<br>1<br>5	38<br>23<br>73<br>67<br>85<br>22<br>77	18<br>10<br>18<br>19<br>17<br>15<br>19	
16.	Piroxicam	0.00913255	6.30103	335V<br>336Q<br>339S<br>341Y	1<br>1<br>1<br>1	38<br>23<br>73<br>67	18<br>10<br>18<br>19	
17.	Rofecoxib	0.22399585	6.30103	499R<br>502A<br>504F<br>513A<br>517L	7<br>1<br>5<br>10<br>4	85<br>22<br>77<br>25<br>9	17<br>15<br>19<br>2<br>2	
18.	SC560	0.0237849	6.39794	335V<br>336Q<br>339S<br>341Y<br>504F	2<br>1<br>2<br>1<br>6	38<br>23<br>73<br>67<br>77	18<br>10<br>18<br>19<br>19	
19.	SC58125	0.01738897	6.431798	499R<br>502A<br>504F	4<br>1<br>4	85<br>22<br>77	17<br>15<br>19	

20.	Vdecoxib	0.7564579	6.522879	178Q	11	38	5
				334Y	1	7	4
				335V	3	38	18
				336Q	3	23	10
				338L	4	24	4
				339S	9	73	18
				341Y	2	67	19
				421A	1	5	4
				499R	13	85	17
				502A	2	22	15
				503I	5	17	5
				504F	7	77	19
				513A	15	25	2
				517L	5	9	2
				75H	12	24	2
				79T	5	10	2
21.	CHEMBL257539	0.1167376	6.522879	106R	7	47	7
				335V	1	38	18
				339S	2	73	18
				341Y	5	67	19
				508M	2	34	8
				512G	7	59	8
				516S	9	43	8
22.	Fenclofenac	0.09343407	6.886057	371Y	5	17	3
				508M	2	34	8
				512G	5	59	8
				516S	3	43	8
23.	Flufenamic acid	0.10528901	7	335V	1	38	18
				339S	2	73	18
				341Y	5	67	19
				371Y	7	17	3
				499R	4	85	17
				502A	2	22	15

				504F	2	77	19	
24.	Flurbiprofen	0.01335906	7	106R	3	47	7	
25.	Ketoprofen	0.02308673	7	335V<br>336Q<br>339S<br>341Y<br>499R<br>502A<br>504F	2<br>1<br>3<br>1<br>2<br>1<br>1	38<br>23<br>73<br>67<br>85<br>22<br>77	18<br>10<br>18<br>19<br>17<br>15<br>19	
26.	Licofelon	0.03997682	7.091515	508M<br>512G<br>516S	2<br>4<br>4	34<br>59<br>43	8<br>8<br>8	
27.	Lumiracoxib	0.02972949	7.30103	106R<br>335V<br>339S<br>341Y	4<br>1<br>1<br>5	47<br>38<br>73<br>67	7<br>18<br>18<br>19	
28.	Mefenamic acid	0.018128	8.02	335V<br>339S<br>341Y<br>499R<br>502A<br>504F	1<br>1<br>5<br>1<br>1<br>1	38<br>73<br>67<br>85<br>22<br>77	18<br>18<br>19<br>17<br>15<br>19	
29.	Niflumic acid	0.23149516	8.060481	178Q<br>334Y<br>335V<br>338L<br>339S<br>341Y<br>421A	7<br>1<br>1<br>3<br>2<br>6<br>1	38<br>7<br>38<br>24<br>73<br>67<br>5	5<br>4<br>18<br>4<br>18<br>19<br>4	

				499R<br>502A<br>503I<br>504F<br>508M<br>512G<br>516S	6<br>2<br>4<br>2<br>4<br>11<br>7	85<br>22<br>17<br>77<br>34<br>59<br>43	17<br>15<br>5<br>19<br>8<br>8<br>8	
30.	Superofen	0.01738897	8.30103	499R<br>502A<br>504F	4<br>1<br>4	85<br>22<br>77	17<br>15<br>19	

Table. S3 Calculation of graphlet signature uniqueness score for COX-2. GSUS = Graphlet Signature Uniqueness Score for ligand j. S_{ij} = i^{th} AA making unique signature with inhibitor j. S_i = Total number of unique signatures made by ith AA with all the ligands S_t = Total number of unique signature made by all ligands with all AAs (Signature Pool). l_i = Number of ligands forming unique signatures with i^{th} AA. L = 30, Total number of ligands used in the dataset.

S.No.	Inhibitor	GSUS	pIC_{50}	Active site residues and the corresponding Signature parameters identified				
				Amino Acid	S_{ij}	S_i	l_i	S_t
1.	2-hydroxy-3-methylbenzoic acid	0.002865	2.33	199T	4	195	30	1201 for each Inhibitor
2.	4-amino-2-hydroxybenzoic acid	0.044757	3.12	106E<br>117E<br>96H<br>203L<br>199T<br>200T<br>97W	1<br>1<br>3<br>1<br>6<br>7<br>1	20<br>20<br>12<br>17<br>195<br>129<br>5	19<br>16<br>3<br>17<br>30<br>17<br>5	
3.	2-hydroxy-5-sulfobenzoic acid	0.047693	3.54	67N<br>106E<br>117E<br>119H<br>203L<br>60L<br>199T<br>200T	10<br>1<br>1<br>6<br>1<br>4<br>7<br>8	155<br>20<br>20<br>87<br>17<br>59<br>195<br>129	15<br>19<br>16<br>14<br>17<br>15<br>30<br>17	
4.	Saccharin	0.009956	5.225483	203L<br>199T<br>200T	1<br>5<br>4	17<br>195<br>129	17<br>30<br>17	
5.	(E)-6-oxo-3-(2-(4-	0.177222	5.35	106E	1	20	19	

	(N-(pyridin-2-yl)sulfamoyl)phenyl)hydrazono)cyclohexa-1,4-dienecarboxylic acid			117E<br>119H<br>203L<br>202P<br>199T<br>200T	1<br>6<br>1<br>7<br>7<br>8	20<br>87<br>17<br>7<br>195<br>129	16<br>14<br>17<br>1<br>30<br>17	
6.	2-hydroxy-3,5-dinitrobenzoic acid	0.016072	5.55	67N<br>60L<br>199T	6<br>3<br>5	155<br>59<br>195	15<br>15<br>30	
7.	3-(4-sulfamoylphenyl)propanoic acid	0.044916	6.305395	67N<br>106E<br>117E<br>119H<br>203L<br>60L<br>199T<br>200T	10<br>1<br>1<br>6<br>1<br>4<br>7<br>8	155<br>20<br>20<br>87<br>17<br>59<br>195<br>129	15<br>19<br>16<br>14<br>17<br>15<br>30<br>17	
8.	2-aminobenzenesulfonamide	0.02644	6.530178	106E<br>117E<br>119H<br>203L<br>199T<br>200T	1<br>1<br>6<br>1<br>6<br>7	20<br>20<br>87<br>17<br>195<br>129	19<br>16<br>14<br>17<br>30<br>17	
9.	4-sulfamoylbenzoic acid	0.105051	6.876148	67N<br>106E<br>117E<br>96H<br>198L<br>203L<br>204L<br>60L<br>199T<br>200T	11<br>1<br>1<br>3<br>9<br>1<br>2<br>4<br>7<br>8	155<br>20<br>20<br>12<br>61<br>17<br>15<br>59<br>195<br>129	15<br>19<br>16<br>3<br>6<br>17<br>6<br>15<br>30<br>17	

				97W	1	5	5	
10.	2-hydrazinylbenzenesulfonamide	0.098203	6.906578	106E<br>117E<br>119H<br>203L<br>197S<br>29S<br>199T<br>200T<br>209W	1<br>1<br>6<br>1<br>6<br>1<br>7<br>8<br>6	20<br>20<br>87<br>17<br>27<br>5<br>195<br>129<br>25	19<br>16<br>14<br>17<br>4<br>4<br>30<br>17<br>4	
11.	4-amino-6-chlorobenzene-1,3-disulfonamide	0.089529	7.124939	67N<br>92Q<br>106E<br>117E<br>119H<br>94H<br>91I<br>203L<br>60L<br>199T<br>200T<br>121V	11<br>10<br>1<br>1<br>6<br>4<br>2<br>1<br>4<br>7<br>8<br>7	155<br>119<br>20<br>20<br>87<br>66<br>23<br>17<br>59<br>195<br>129<br>78	15<br>12<br>19<br>16<br>14<br>12<br>12<br>17<br>15<br>30<br>17<br>12	
12.	4-amino-6-(trifluoromethyl)benzene-1,3-disulfonamide	0.046369	7.200659	67N<br>106E<br>117E<br>119H<br>203L<br>60L<br>199T<br>200T	9<br>1<br>1<br>6<br>1<br>4<br>7<br>8	155<br>20<br>20<br>87<br>17<br>59<br>195<br>129	15<br>19<br>16<br>14<br>17<br>15<br>30<br>17	
13.	4-amino-3-fluorobenzenesulfonamide	0.02644	7.221849	106E<br>117E	1<br>1	20<br>20	19<br>16	

				119H<br>203L<br>199T<br>200T	6<br>1<br>6<br>7	87<br>17<br>195<br>129	14<br>17<br>30<br>17	
14.	4-amino-N-(4-sulfamoylphenethyl) benzenesulfonamide	0.148955	7.30103	65A<br>62N<br>67N<br>106E<br>117E<br>119H<br>203L<br>60L<br>95F<br>199T<br>200T	3<br>7<br>11<br>1<br>1<br>6<br>1<br>4<br>8<br>7<br>8	12<br>31<br>155<br>20<br>20<br>87<br>17<br>59<br>24<br>195<br>129	5<br>5<br>15<br>19<br>16<br>14<br>17<br>15<br>3<br>30<br>17	
15.	Methazolamide	0.114469	7.30103	67N<br>92Q<br>94H<br>91I<br>198L<br>203L<br>204L<br>60L<br>199T<br>200T<br>121V	11<br>10<br>4<br>2<br>9<br>1<br>2<br>4<br>6<br>8<br>7	155<br>119<br>66<br>23<br>61<br>17<br>15<br>59<br>195<br>129<br>78	15<br>12<br>12<br>12<br>6<br>17<br>6<br>15<br>30<br>17<br>12	
16.	4-amino-N-(4-sulfamoylbenzyl)be nzenesulfonamide	0.137639	7.337242	65A<br>62N<br>67N<br>106E<br>117E<br>119H<br>60L<br>95F<br>199T	3<br>7<br>11<br>1<br>1<br>6<br>4<br>8<br>7	12<br>31<br>155<br>20<br>20<br>87<br>59<br>24<br>195	5<br>5<br>15<br>19<br>16<br>14<br>15<br>3<br>30	

17.	Sulpiride	0.097432	7.39794	67N<br>92Q<br>106E<br>117E<br>119H<br>94H<br>91I<br>60L<br>199T<br>121V	11<br>10<br>1<br>1<br>6<br>4<br>2<br>4<br>7<br>7	155<br>119<br>20<br>20<br>87<br>66<br>23<br>59<br>195<br>78	15<br>12<br>19<br>16<br>14<br>12<br>12<br>15<br>30<br>12	
18.	Dichlorophenamide	0.076751	7.420216	67N<br>92Q<br>94H<br>91I<br>203L<br>60L<br>199T<br>200T<br>121V	11<br>10<br>4<br>2<br>1<br>4<br>6<br>8<br>7	155<br>119<br>66<br>23<br>17<br>59<br>195<br>129<br>78	15<br>12<br>12<br>12<br>17<br>15<br>30<br>17<br>12	
19.	Zonisamide	0.055204	7.455932	92Q<br>106E<br>117E<br>119H<br>94H<br>91I<br>199T<br>121V	9<br>1<br>1<br>6<br>4<br>2<br>6<br>7	119<br>20<br>20<br>87<br>66<br>23<br>195<br>78	12<br>19<br>16<br>14<br>12<br>12<br>30<br>12	
20.	4-((2-aminopyrimidin-4-yl)amino)benzenesulfonamide	0.162283	7.481486	67N<br>203L<br>60L<br>95F<br>197S<br>29S<br>199T<br>200T	11<br>1<br>4<br>8<br>6<br>1<br>6<br>8	155<br>17<br>59<br>24<br>27<br>5<br>195<br>129	15<br>17<br>15<br>3<br>4<br>4<br>30<br>17	

				209W	6	25	4	
21.	Celecoxib	0.088067	7.677781	67N<br>60L<br>197S<br>29S<br>199T<br>209W	10<br>4<br>4<br>1<br>5<br>6	155<br>59<br>27<br>5<br>195<br>25	15<br>15<br>4<br>4<br>30<br>4	
22.	5-imino-4-methyl-4,5-dihydro-1,3,4-thiadiazole-2-sulfonamide	0.095185	7.721246	65A<br>62N<br>67N<br>106E<br>117E<br>119H<br>64H<br>203L<br>60L<br>199T<br>200T<br>97W	1<br>3<br>11<br>1<br>1<br>6<br>3<br>1<br>4<br>7<br>8<br>1	12<br>31<br>155<br>20<br>20<br>87<br>7<br>17<br>59<br>195<br>129<br>5	5<br>5<br>15<br>19<br>16<br>14<br>3<br>17<br>15<br>30<br>17<br>5	
23.	Indisulam	0.082407	7.823909	65A<br>62N<br>67N<br>64H<br>203L<br>60L<br>199T<br>200T<br>97W	1<br>3<br>11<br>3<br>1<br>4<br>6<br>8<br>1	12<br>31<br>155<br>7<br>17<br>59<br>195<br>129<br>5	5<br>5<br>15<br>3<br>17<br>15<br>30<br>17<br>5	
24.	Acetazolamide	0.0338	7.920819	92Q<br>94H<br>91I<br>199T	6<br>3<br>2<br>5	119<br>66<br>23<br>195	12<br>12<br>12<br>30	

				121V	6	78	12	
25.	Topiramate	0.3007	8	65A<br>62N<br>92Q<br>106E<br>64H<br>94H<br>91I<br>198L<br>204L<br>197S<br>29S<br>199T<br>209W<br>121V	4<br>11<br>16<br>1<br>1<br>9<br>2<br>13<br>3<br>10<br>2<br>10<br>7<br>7	12<br>31<br>119<br>20<br>7<br>66<br>23<br>61<br>15<br>27<br>5<br>195<br>25<br>78	5<br>5<br>12<br>19<br>3<br>12<br>12<br>6<br>6<br>4<br>4<br>30<br>4<br>12	
26.	Sulthiame	0.03563	8.045757	92Q<br>94H<br>91I<br>199T<br>121V	6<br>3<br>2<br>5<br>6	119<br>66<br>23<br>195<br>78	12<br>12<br>12<br>30<br>12	
27.	Benzolamide	0.076824	8.045757	92Q<br>94H<br>91I<br>198L<br>204L<br>199T<br>121V	9<br>4<br>2<br>8<br>2<br>5<br>7	119<br>66<br>23<br>61<br>15<br>195<br>78	12<br>12<br>12<br>6<br>6<br>30<br>12	
28.	Dorzolamide	0.110511	8.045757	92Q<br>106E<br>94H<br>91I<br>198L<br>204L<br>199T	14<br>1<br>8<br>2<br>11<br>3<br>9	119<br>20<br>66<br>23<br>61<br>15<br>195	12<br>19<br>12<br>12<br>6<br>6<br>30	

				121V	7	78	12	
29.	Ethoxzolamide	0.16463	8.09691	244N	1	1	1	
				92Q	5	119	12	
				106E	2	20	19	
				117E	5	20	16	
				107H	1	1	1	
				119H	9	87	14	
				94H	11	66	12	
				96H	6	12	3	
				91I	1	23	12	
				199T	8	195	30	
				97W	1	5	5	
				121V	3	78	12	
30.	Brinzolamide	0.110511	8.522879	92Q	14	119	12	
				106E	1	20	19	
				94H	8	66	12	
				91I	2	23	12	
				198L	11	61	6	
				204L	3	15	6	
				199T	9	195	30	
				121V	7	78	12	

Table. S4 Calculation of graphlet signature uniqueness score for CA-II. GSUS = Graphlet Signature Uniqueness Score for ligand j. S_{ij} = ith AA making unique signature with inhibitor j. S_i = Total number of unique signatures made by ith AA with all the ligands S_t = Total number of unique signature made by all ligands with all AAs (Signature Pool). l_i = Number of ligands forming unique signatures with ith AA. L =30, Total number of ligands used in the dataset.

Compound Name	Similarity	IC50	pIC50	GSUS	Docking score
6MMPA	0.789474	80000	4.09691	0.16908121	-7.09
Ibuprofen	0.789474	40000	4.39794	0.01738897	-7.04
6MMPA	0.738095	80000	4.09691	0.16908121	-7.09
Naproxen	0.738095	2000	5.69897	0.05585896	-7.15
Celecoxib	0.72973	50	7.30103	0.40196448	-10.35
SC-560	0.72973	6300	5.200659	0.0237849	-8.74
Celecoxib	0.864286	50	7.30103	0.40196448	-10.35
SC58125	0.864286	300	6.522879	0.01738897	-9.99
SC560	0.724832	6300	5.200659	0.0237849	-8.74
SC-58125	0.724832	300	6.522879	0.01738897	-9.99
Dichlofenac	0.764045	9.4	8.02	0.44306776	-8.32

Lumiracoxib	0.764045	7000	5.154902	0.02972949	-7.68
Flufenamic acid	0.830769	20000	4.69897	0.10528901	-7.1
Mefenamic acid	0.830769	300	6.522879	0.018128	-7.56
Piroxicam	0.758065	70000	4.154902	0.00913255	-8.13
Meloxicam	0.758065	400	6.39794	0.49026752	-8.27
Nimuslide	0.727273	500	6.30103	0.06857699	-8.98
NS398	0.727273	81	7.091515	0.05656345	-9.1

Table S5. Similar compounds with different activity in COX-2.(Comparison of GSUS and Autodock score)

Compound Name	Similarity	IC_{50}	pIC_{50}	GSUS	Docking score
CHEMBL257539	0.277108	100	7	0.1167376	-8.65
Indisulam	0.277108	100	7	0.12526685	-9.46
CHEMBL257539	0.17931	100	7	0.1167376	-8.65
Niflumic acid	0.17931	100	7	0.23149516	-6.67
Indisulam	0.24581	100	7	0.12526685	-9.46
Niflumic acid	0.24581	100	7	0.23149516	-6.67
SC58125	0.154321	300	6.522879	0.01738897	-9.99
Mefenamic acid	0.154321	300	6.522879	0.018128	-7.56
Flurbiprofen	0.065574	500	6.30103	0.01335906	-7.58
Nimuslide	0.065574	500	6.30103	0.06857699	-8.98
Flurbiprofen	0.297297	500	6.30103	0.01335906	-7.58
RFXCB	0.297297	500	6.30103	0.22399585	-10.79
Nimuslide	0.118421	500	6.30103	0.06857699	-8.98
Rofecoxib	0.118421	500	6.30103	0.22399585	-10.79

Table S6. Compounds with similar activity but different structure in COX-2. (Comparison of GSUS and Autodock score).

Compound Name	Similarity	IC_{50}(nM)	pIC_{50}	GSUS	Docking score
2-aminobenzenesulfonamide	0.7907	295	6.530178	0.02644	-5.75
2-hydrazinylbenzenesulfonamide	0.7907	124	6.906578	0.098203	-6.21
2-hydroxy-3-methylbenzoic acid	0.7174	4700000	2.33	0.002865	-5.08
4-amino-2-hydroxybenzoic acid	0.7174	750000	3.12	0.044757	-4.6
4-amino-6-chlorobenzene-1,3-disulfonamide	0.7419	75	7.124939	0.089529	-7.07
Dichlorophenamide	0.7419	38	7.420216	0.076751	-5.38

Table S7. Similar compounds with different activity in CA-II. (Comparison of GSUS and Autodock score)

Compound Name	Similarity	IC_{50}(nM)	pIC_{50}	GSUS	Docking score
Sulthiame	0.237179	9	8.045757	0.03563	-4.39
Benzolamide	0.237179	9	8.045757	0.076824	-5.06
Sulthiame	0.21267	9	8.045757	0.03563	-4.39
Dorzolamide	0.21267	9	8.045757	0.110511	-5.69
Benzolamide	0.1841	9	8.045757	0.076824	-5.06
Dorzolamide	0.1841	9	8.045757	0.110511	-5.69
Dorzolamide	0.188285	9	8.045757	0.110511	-5.69
Ethoxzolamide	0.188285	8	8.09691	0.16463	-5.18
Acetazolamide	0.0625	12	7.920819	0.0338	-4.66
Topiramate	0.0625	10	8	0.3007	-4.86
Celecoxib	0.120879	21	7.677781	0.088067	-6.56
5-imino-4-methyl-4,5-dihydro-1,3,4-thiadiazole-2-sulfonamide	0.120879	19	7.721246	0.095185	-5.35
Zonisamide	0.135135	35	7.455932	0.055204	-6.95
4-((2-aminopyrimidin-4-yl)amino)benzenesulfonamide	0.135135	33	7.481486	0.162283	-5.79
Dichlorophenamide	0.15	38	7.420216	0.076751	-5.38
Zonisamide	0.15	35	7.455932	0.055204	-6.95

Table S8. Compounds with similar activity but different structure in CA-II. (Comparison of GSUS and Autodock score)
